# The evolving culture of medical cannabis in Canada for the management of chronic pain

**DOI:** 10.3389/fphar.2023.1153584

**Published:** 2023-04-07

**Authors:** H. Clarke, M. Fitzcharles

**Affiliations:** ^1^ Department of Anesthesiology and Pain Medicine, University of Toronto, Toronto, ON, Canada; ^2^ Department of Anesthesia and Pain Management Pain Research Unit, Toronto General Hospital, Toronto, ON, Canada; ^3^ Transitional Pain Service, Toronto General Hospital, Toronto, ON, Canada; ^4^ Department of Rheumatology, McGill University, Montreal, QC, Canada; ^5^ Alan Edwards Pain Management Unit, McGill University, Montreal, QC, Canada

**Keywords:** medical, cannabis, legalization, chronic, pain

## Abstract

Although used therapeutically for millennia, cannabis has been a prohibited substance worldwide for most of the 20th Century. With revision of prohibitive regulations in many jurisdictions during the past 2 decades, cannabis is increasingly available to patients as a potential treatment option for various symptoms. Pain relief, sleep promotion and alleviation of distress, depression and anxiety are the most common reasons for cannabis use. Canada has been at the forefront of medical cannabis (MC) legislation revisions to enable and facilitate access for therapeutic use. Although initially viewed with caution and stigma, attitudes to cannabis in general have changed. Medical cannabis is identified as the herbal plant product sourced from a grower/producer and is not at present a regulated pharmaceutical product. Medical cannabis use is currently prevalent in Canada but has bypassed the rigorous study required for usual drug approval. Although uptake has been enthusiastic by patients, the medical community has voiced cautions and concerns. Access to medical cannabis is fairly easy once an approval document is obtained from a healthcare professional, but without obligation for medical or pharmacy oversight. The greatest concern is a dearth of sound clinical evidence for effects and harms. Emerging concerns include prevalent patient self-management with information based on personal research, an abundance of on-line information which may not always be accurate, the emergence of designated “cannabis clinics,” potential risks to society due to accidents, and high cost of the legal medical product leading to access *via* the recreational market. With cannabis now entrenched in Canadian healthcare, physicians must be sufficiently knowledgeable to provide guidance that is evidence-based and will ensure personal and societal harm reduction. Examination of the changing culture of medical cannabis in Canada will provide insight for countries that may be anticipating similar revisions of cannabis regulations to allow cannabis access for their patient population and learn from the issues created by recreational legalization.

## 1 Introduction

Cannabis has been used as a medicinal product for over 5,000 years with earliest written records found in papyrus writings from Egypt in about 1500 BC, on Assyrian clay tablets a century later, and in a compendium of medicinal herbs written during the Han dynasty in China (221 BC to AD 220) (Crocq, 2020). These early writings refer to cannabis as an agent to relieve pain and inflammation, but also to promote “delight” (for Scythians according to Herodotus c.484-c.425 BC), with topical, oral or inhalation methods of administration. Medical use has subsequently been documented through the ages with more prevalent use in Western medicine in the 19th century. Although the therapeutic effects have been claimed for various conditions for centuries, the psychoactive effects leading to widespread recreational use caused cannabis to fall into disfavour (Kalant, 2001). From the early 1900s and beginning in the United States (US), legislation has progressively limited access to cannabis. According to the 1961 United Nations Single Convention on Narcotic Drugs, a treaty with the aim to combat drug abuse by a coordinated international action, cannabis regulations were similar to those applied to other narcotic drugs (United_Nations, 1961). Once identified as a prohibited substance, clinical use and research into effects of cannabis was not possible. Due to strong public advocacy, the past 2 decades have seen a progressive change in regulations in many countries allowing for increased access to cannabis for medical purposes. With promise of effect on an array of symptoms, including pain relief, sleep promotion, and help with mental health issues, the increased availability of medical cannabis (MC), that is cannabis legally available for medicinal purpose, has been enthusiastically welcomed by the patient community, but with cautions expressed by healthcare practitioners. There are currently ongoing studies examining the effects of cannabinoids as a specific treatment strategy for various conditions, e.g., epilepsy, autism, headaches, opioid use disorder, etc (Vimal et al., 2023). Medical legalization followed by decriminalization and eventual recreational legalization has permitted the culture of MC to evolve. This narrative review will focus on the use of MC, identified as the herbal plant product sourced from a grower/producer and not a regulated pharmaceutical product, for the management of chronic pain, examine the current status of MC in North America (with particular attention to Canada) from both the patient and healthcare perspective, and offer suggestions on future directions.

## 2 The evolving legal framework for medical cannabis and its source and price

Beliefs, rather than evidence from scientific study, that cannabis has medical benefits has influenced legislators worldwide to debate the merits of legalizing cannabis for medicinal use with an evolution of this legal landscape over the past 2 decades (Sznitman and Bretteville-Jensen, 2015). Driven by public advocacy, regulators first in the United States (US) and then Canada loosened cannabis regulations to allow access for medical use. Beginning with California in 1996, there has been legalization of MC in various US states, and legal access for Canadian patients since July 2001 (Health_Canada, 2001; Orenstein and Glantz, 2020). Cannabis is currently available as a medical product in 37 of 50 US states, and as a recreational product in 19 US states. Since October 2018, cannabis is available as a recreational product for adults in Canada.

Two pharmaceutical cannabinoid products are approved by Health Canada, Sativex (pure THC/CBD extract) and nabilone (a synthetic CB1 agonist). The specific Health Canada labelled indications for these two products are for adult patients with moderate to severe spasticity due to multiple sclerosis and chemotherapy induced nausea and vomiting respectively. Therefore, access for patients with chronic pain is considered off-label use. As drug formulary directives are under the jurisdiction of individual provinces, off-label access and insurance coverage can differ between provinces. In the absence of insurance, either public or private, use is limited by cost rather than patient preference or response to treatment.

MC has followed an unconventional path for introduction of a therapeutic product by bypassing the usual drug approval processes in Canada and the United States. In these countries the regulatory authorities have allowed patient access to MC by the simple process of authorization for use by a physician or other healthcare professional (in Canada), rather than following the standard procedure of a prescribed medication that is dispensed by a pharmacist. In Canada the authorization document can currently be presented to a cannabis producer or a cannabis dispensary with no further obligation for medical care during the period of MC authorization, which may be up to 1 year. This breach in medical care has prompted various physician and medical groups to strongly recommended appropriate medical follow-up when patients use MC (Fitzcharles et al., 2019; Petzke et al., 2019; Chang-Douglass et al., 2020; Bhaskar et al., 2021; Busse et al., 2021).

### 2.1 Timeline for access to medical cannabis in Canada

As Canada has been at the forefront of cannabis legalization, the timeline for access will be examined. Although MC is not a Health Canada approved treatment, legislation has progressively allowed for easier access for patients. Following court rulings based on appeals by patient advocates, Health Canada adopted the *Marijuana Medical Access Regulations* (MMAR) in 2001 which allowed seriously ill Canadians to obtain authorization to possess dried marijuana for their own medical use with the authorization of a medical practitioner. Certain medical conditions were allowed and included *severe pain from severe forms of arthritis*, when the medical practitioner stated that “conventional treatment(s) have been tried or considered and have been found to be ineffective or medically inappropriate”. Under these regulations, the medical practitioner provided the medical justification for an application but did not provide a traditional prescription. Approval for possession of a specified amount of herbal cannabis was given by Health Canada through the *Medical Marijuana Access Regulations* (MMAP) for a period up to 1 year. Once approved, Health Canada arranged for cannabis to be sent directly to the patient (Health Canada, 2010). These initial regulations were eased when Health Canada repealed the MMAR in March 2014 and replaced the MMAR with the “*Marijuana for Medical Purposes Regulations*” (MMPR) (Government_of_Canada, 2013). Under these regulations the medical practitioner is required to complete a “medical document”, stating the daily dose and duration of use, without restriction for specified medical conditions, and without need to document failure of conventional treatments. This was met with mixed emotions from healthcare practitioners, most of whom resisted or refused any involvement in medical authorization of cannabis, while a small minority embraced cannabis authorization and created clinics often associated with Licensed Producers. Cannabis medical legalization and its evolution over the past decades has promoted the perception of safety compared to traditional drug therapies for many conditions and has influenced uptake of cannabis by adults (Carliner et al., 2017).

### 2.2 Effect of recreational cannabis legalization on sourcing and pricing

Recreational cannabis legalization in Canada in 2018 has allowed adults easy access *via* the commercial route (Cox, 2018; Government_of_Canada). This has in turn influenced MC use by attenuating the stigma associated with a previously illegal recreational product and by increasing social acceptability. Furthermore, patients may now circumvent the administrative burden of medical authorization and self-experiment without medical oversight. MC is costly and mostly not reimbursed by public or private insurers (except for Veterans Affairs), leading many to turn to the recreational market, and more specifically the illegal street market (Cannabisbenchmarks, 2023). In a study of rheumatology attendees in Montreal, Canada in June to August 2019, only 20% of current medical users purchased cannabis entirely *via* the legal medical route, with 40% obtaining access *via* illegal avenues, and the remaining by commercial or on-line purchase (Fitzcharles et al., 2020). Anecdotally, some patients report home preparations of edible products such as cannabis-based butters or cookies, the practice of which is discouraged by the medical community as ingestion of product in this way detracts from the concept of a medicinal therapeutic intervention. It is encouraging to note that Canadians are slowly moving towards the legal market which has some regulatory control of quality production to access cannabis. The findings of an on-line study of cannabis consumers at two timepoints, 2019 and 2020, after legalization in Canada, were that consumers increasingly purchased dried flower cannabis from the legal source compared to the illegal source (45.7% vs. 58.1%) (Statcan, 2021; Wadsworth et al., 2022). Recreational cannabis legalization has been associated with decreased cost of legal dried flowers in 2020 compared to 2019 ($12.63 vs $11.16; *p*˂0.001), with legal cannabis more expensive than illegal cannabis in both years ($12.63 vs. $9.04 in 2019; *p* < 0.001, $11.16 vs. $9.41 in 2020; *p* < 0.001) (Wadsworth et al., 2022). Recreational legalization has promoted the production of an array of various cannabis-based products, including edibles and infused drinks, that are available *via* both the legal and illegal market. Although cannabis edibles may be legal federally, provincial and territorial governments regulate the rules that govern the sale and public consumption of these edibles. As noted above, this method of administration by patients should be discouraged. When a product is used therapeutically, patients should be aware of the dosing as applies to all medications. It is hoped that price and retail policies will continue to encourage the transition to the safer legal market, albeit not the ideal medical route. These combinations of factors have contributed to increased patient acceptance and also self-medication with cannabis (Hall et al., 2019).

## 3 Challenges from the healthcare perspective

MC has been accepted by Canadian patients suffering from conditions that cause pain such as headache, osteoarthritis, cancer and multiple sclerosis (Banwell et al., 2016; Baron et al., 2018; McTaggart-Cowan et al., 2021). Survey studies have consistently reported positive attitudes of the public towards cannabis as a therapy, with the perception that MC is a neglected natural and safe treatment alternative for many symptoms, but with more circumspection expressed by the healthcare community (Carliner et al., 2017; Cheng et al., 2022; D'Souza and Ranganathan, 2015). Based on the Health Canada cannabis survey, people felt that occasional use of alcohol or cannabis had no or only slight risk. Whereas the majority of respondents deemed smoking tobacco and using an e-cigarette with nicotine occasionally as having a moderate or great risk (Canadian_Cannabis_Survey, 2021). In this survey, 50% of those using cannabis for medical purposes reported use of dried flower/leaf as combustible and vaporized forms, which represent the bulk of products consumed by cannabis users, which pose a more significant health risk than non-combustible forms.

The healthcare community continue to voice significant concerns regarding limited evidence for efficacy, concerns about immediate and long-term harms, societal implications for potential harms, and the overabundance of media and on-line coverage as well as marketing campaigns. The National Academies of Sciences, Engineering and Medicine published a comprehensive document in 2017 summarizing current evidence for therapeutic use of MC, highlighting the areas of limited evidence for effect in management of chronic pain (National Academies of Sciences, 2017). Although this document was published 5 years ago, there has been little new published information on MC effects in chronic pain. Attitudes and perspectives of healthcare professionals regarding MC for chronic pain were analysed in a systematic review of 26 studies involving countries in North America, Europe, Israel and Australia (Cheng et al., 2022). Themes that emerged included legal issues, low perceived knowledge and need for professional education, issues of addiction and abuse, and comparative safety of MC compared to opioids. These themes underline the need for education of healthcare professionals and the development of evidence-based guidelines to address efficacy, safety and appropriate doing of MC for chronic pain (Cheng et al., 2022).

### 3.1 Evidence for effect of cannabis for chronic pain

Clinical evidence needed for widespread acceptance and implementation of a drug for a condition specific indication is accrued *via* the successful completion of randomized controlled clinical trials (RCTs). This information is lacking for MC due to two barriers: 1) few formulations of plant based medical cannabis extracts in the North American marketplace meet pharmaceutical standards required for Health Canada/Food and Drug Administration (FDA) regulatory approval; 2) the designation of cannabis (in particular THC) as a controlled substance. These barriers have contributed to a paucity of clinical trial literature, especially for management of chronic pain especially of a musculoskeletal nature, with current evidence deemed insufficient to justify use (Fitzcharles et al., 2016). The importance of the placebo effect in studies of cannabis-based therapies for chronic pain has been highlighted by the results of a systematic review and meta-analysis of twenty studies, including 1,459 individuals (Gedin et al., 2022). Pain intensity was significantly reduced in response to placebo, with a moderate to large effect size [mean (SE) Hedges g, 0.64 (0.13); *p* < 0.001], leading the authors to conclude that placebo contributes significantly to pain reduction, and postulating that positive media attention may have played a role in the drugs acceptance and popularity among patients. Given the absence of clinical evidence, physicians have lacked confidence in their knowledge of cannabis as a therapy and continue to be hesitant to recommend its use (Fitzcharles et al., 2014; Ablin et al., 2016; Rønne et al., 2021; Cheng et al., 2022). In Canada, only about 8% of the 92,173 registered physicians provided medical authorization for MC in 2021, but with considerable differences noted between the various provinces. It is therefore understandable that patients wishing to try MC may choose to seek other avenues of access which often leaves them vulnerable to poor outcomes.

### 3.2 Increase in self-administration

Cannabis recreational legalization in Canada has opened an avenue for easier access for patients wishing to self-medicate or experiment with use, but often without physician or pharmacist oversight. Patients can thus avoid tensions or conflicts with physicians unwilling to authorize use and by-pass the administrative steps to access MC. Although many obtain cannabis from legal recreational outlets, the more favourable pricing of street cannabis is a necessity for many patients. Since cannabis recreational legalization, only 20% of surveyed Canadian rheumatology patients accessed MC entirely *via* the legal medical route, with many obtaining cannabis for therapeutic reasons *via* the illegal recreational route (Fitzcharles et al., 2020). Of concern is that only one-third disclosed their cannabis use to their treating physician. Given this increase in acceptance post legalization and the ease of access, the 2021 Canadian Cannabis survey identified that 25% of Canadians have used cannabis in the last 12 months with the younger demographic (<25) approximately doubling that of 25 and older Canadians (Canadian_Cannabis_Survey, 2021). This pattern of accessing cannabis illegally for medical purposes is seen similarly in other countries. In an anonymous on-line survey of therapeutic cannabis use in Australia, only 2.7% of 1,388 respondents accessed cannabis *via* the legal medical route and only 5% sought advice about cannabis from a healthcare professional, often relying on personal study, internet-based media or cannabis advocacy groups (Lintzeris et al., 2020). The main perceived barriers to MC use reported in this study were cost, disinterest from the medical profession and stigma. Street cannabis use by patients raises concerns about molecular content of Δ9-tetrahydrocannabinol (THC) and cannabidiol (CBD), quality, additive products, contaminants and the increasing concentrations of THC. Canada has implemented stringent quality control and quality assurance measures for all classes of cannabis to sufficiently inform consumers and to ensure that the cannabis supply is safe (Pusiak et al., 2021). However these regulatory standards fall short compared to tobacco regulations (Moir et al., 2008). We note that mean concentrations of THC in illicit cannabis have steadily increased globally, with levels almost doubling from around 9% in 2,000% to 18% in a period of 5 years as noted in the Netherlands (Mensinga et al., 2006). The THC content of cannabis products offered by on-line dispensaries in the US did not differ between recreational or medical use, and for medical use was on the higher side at about 20% (Cash et al., 2020). Studies of MC to date have examined effects of cannabis with THC content mostly in the order of 9%, with a single study in pain relief using 12.5% THC content (Ware et al., 2015).

### 3.3 Potential risks associated with medical cannabis

Patients may not be sufficiently aware of risks associated with cannabis, especially when consumed for a health-related condition without supervision. The potential risks can extend beyond the individual and have societal implications such as occurs when persons experience psychomotor effects and are at risk of motor vehicle accidents (Asbridge et al., 2005; Lee et al., 2021). In a study assessing the impact of MC authorization on motor vehicle-related health utilization visits in Ontario, Canada, between 2014 and 2017, Lee et al. reported an increase of 2.92 events/10,000 (95%CI 0.64–5.19) compared to controls over the follow-up period. This effect was largely driven by motor vehicle crash related emergency department visits (+0.80 events/10,000, *p* < 0.001) (Lee et al., 2021).

The long-term effects of cannabis must be considered, especially for a younger person using MC for treatment of chronic pain, with the potential for a daily lifelong treatment. There is little debate that the daily use of high dose THC products increase the risk of developing a cannabis use disorder. The 3 year incidence of cannabis dependence for frequent cannabis users is reported as 37% (van der Pol et al., 2015). In the coming years, it will be important for data to continue to be collected on the amount of cannabis used, whether higher concentrations of THC is favoured and the incidence of cannabis use disorder for patients using MC in Canada.

### 3.4 Medical cannabis as a substitute for opioids

Can MC be used as an effective substitute for opioids in persons with chronic pain with less potential for harm? Opioid reduction has been commonly reported in both on-line and observational studies, but with lesser effects observed in the setting of formal controlled trials (Lucas and Walsh, 2017; Abuhasira et al., 2018; Boehnke et al., 2019b; Meng et al., 2021; Noori et al., 2021). Almost half of 2,697 Canadian participants in an on-line survey receiving legal MC reported that cannabis had enabled substitution for other substances (alcohol 18%, tobacco 8%, opioids 18%, other prescription medications 18%) (Holman et al., 2022). An observational study of 757 patients followed at community-based cannabis clinics in Ontario, Canada reported that the proportion of those using opioids decreased by half from 41% to 24% at 12 months (Meng et al., 2021). In a small cohort of chronic pain patients consuming cannabis, the majority (96%) self-reported effective pain management, and 76% reported a significant decrease in analgesic medication usage (Ajrawat et al., 2022). Similarly, over half of participants with rheumatic diseases in a survey of Canadian and US participants reported discontinuation of various medication groups, including opioids as a result of MC substitution (Boehnke et al., 2022). In an on-line survey of over 800 persons with fibromyalgia, 53% reported that cannabidiol (CBD) products allowed them to reduce or even discontinue opioid use (Boehnke et al., 2021).

In contrast to the encouraging reports of opioid reduction in cohort studies, the conclusions of a systematic review and meta-analysis which included five RCTs (patients with cancer-related pain) and 12 observational studies (10 included patients with chronic non-cancer pain) are that the opioid-sparing effects of cannabinoids for chronic pain remains uncertain due to very low certainty evidence (Noori et al., 2021). However, one of the major caveats is that patients included in the RCTs were instructed not to alter their opioid dose, as such the expectation of an opioid reduction seems unrealistic. Non-etheless the authors reported that the addition of cannabinoids resulted in only a trivial difference in cancer-related pain (weighted mean difference (WMD) −0.18 cm; 95%CI −0.38–0.02 on the 10 cm VAS for pain), and very low certainty evidence for opioid reduction among patients with chronic non-cancer pain (WMD −22.5 mg morphine equivalent (MME); 95%CI −43.06–−1.97) (Noori et al., 2021). In 2021, using Delphi methodology, a group of experts proposed a strategy for the safe introduction and titration of cannabinoids in concert with opioid tapering (Sihota et al., 2021).

### 3.5 Cannabis clinics and dispensaries

As the cannabis industry flourished from 2014—2018, many specialized “cannabis clinics” staffed by healthcare professionals emerged. These clinics were often developed to fill a void in patient care as many physicians may have been unwilling to prescribe cannabinoids either due to lack of confidence in their personal knowledge, concerns about the evidence for effect, or simply personal bias. When functioning only to prescribe cannabis, these clinics represent less than ideal medical care, but did provide a service that should not have been necessary. It is however notable that the primary purpose and *raison d’ etre* for a cannabis clinic is the authorization of cannabis products for health-related conditions. Some clinics took a wholistic approach, but others were less committed to comprehensive patient care, and were primarily focused on the authorization of cannabis and often associated with particular licenced producers. One-third of patients who responded to an on-line survey and were Canadian federally-authorized cannabis patients received their authorized products from a secondary specialized cannabis care provider, most of whom worked in a clinic that specialized in MC (Holman et al., 2022). Although participants rated their primary care provider as having “good” knowledge of MC and were moderately confident that MC could be integrated into their treatment, higher ratings were given for secondary care providers knowledge about MC, but with 71% of secondary providers not involved in the participants’ medical care other than authorizing MC (Holman et al., 2022).

It is unique and outside the usual paradigm of medical care that a physician should focus on the prescription of a single drug. The Canadian Medical Protective Association, the largest medical insurance liability provider in Canada, has cautioned physicians to only provide a prescription for MC when conventional treatments have failed or are inappropriate, when “they have the necessary clinical knowledge to engage in meaningful consent discussions with patients” and should inform the patient of “the lack of information to date.” (Canadian_Medical_Protective_Association). Even prior to the advent of virtual medicine as a new form of care introduced in early 2020 during the COVID pandemic, many cannabis clinics offered an on-line consultation for the purpose of providing authorization for MC with only superficial knowledge of the patient. Cannabis clinics advertise their services as “experts” in this field which is a misrepresentation to a patient community that requires comprehensive clinical care and not a single magic potion.

Cannabis dispensary staff and licensed producer personnel have become increasingly present in counselling patients on use of MC, often without medical background and therefore at risk of providing inappropriate medical advice (Dickson et al., 2018; Lim and Kirchhof, 2019; Merlin et al., 2021). In a US survey of 434 cannabis dispensary staff, with only 13% identified as pharmacists, advice about MC was given mostly based on the medical condition 74%, experience reported by other customers 70%, and dispensary staff personal experience 63%, but with “clinical input” (not further specified) identified for 40% (Merlin et al., 2021). It is also notable that 64% of respondents held a medical cannabis card. It is encouraging to note that cannabis retail workers who recently participated in a focus group study in Washington State identified their professional roles as being compliant with state law and regulations, did not believe their job involved discussions around pregnancy or driving, and educated consumers on how to avoid over intoxication (Carlini et al., 2022). On-line information on MC, especially from dispensaries reflects positive attitudes and beliefs that could be misleading for patients. Cannabis dispensaries are not altruistic health focussed endeavours, but commercial enterprises with the objective of achieving profit by sale. It is therefore not surprising that patients report satisfaction with dispensary interaction (Capler et al., 2017). Furthermore, the greater availability of cannabis dispensaries may be a risk for adverse effects on health, with increasing cannabis dispensary density associated with more hospitalizations for cannabis use disorder in California (Mair et al., 2021). It is encouraging to note that a 2021 survey of dispensary staff in Canada mostly advised against use of MC in pregnancy (Vastis et al., 2021).

The existence of cannabis clinics is dependent on the continued use of MC by those attending. We express concerns that patients may not be receiving the best care for their chronic pain management if the dispensary or the cannabis clinic is their primary pain care provider. Patients should not be receiving medical advice from dispensary staff, with the exception of qualified pharmacists who may be working in a dispensary. We acknowledge that there will be patients who experience symptom relief with use of MC, but this treatment should be a component of a multidisciplinary care model, including non-pharmacologic, as well as other pharmacologic treatments.

### 3.6 Research challenges

Research addressing the effects of MC has had challenges at multiple levels. Initially, any study of cannabis was marred by stigma and the classification of the cannabis plant as a scheduled product/illegal substance. There are currently two Health Canada authorized pharmaceutical cannabis-based medicines available in Canada, Sativex (pure THC/CBD extract) and nabilone (a synthetic CB1 agonist), with specific labelled indications. In order to enable safe integration of plant-based cannabis products into the Canadian marketplace, the Office of Medical Cannabis was created.

Permission to conduct basic and clinical research with cannabis in Canada is complex and requires approval from three different regulatory bodies. In the first instance, clinical trials are regulated in accordance with the *Food and Drugs Act* (FDA), which requires an initial submission to Health Canada which includes a protocol, information on product chemistry and manufacturing data, preclinical study, good manufacturing practices (GMP) and investigators brochure. Thereafter, the FDA requires a “no objection letter” from the office of Clinical Trials in Health Products and Food Branch, and under the *Cannabis Act,* a research licence must be obtained from the Controlled Substance and Cannabis Branch. Beyond this cumbersome application process, clinical research with cannabis products is hampered by the need for product specific GMP, preclinical studies, and investigators brochure with information on dosing and safety, much of which does not exist for individual products. Any basic biomedical research, or even research on the plant itself requires that a licence is obtained and applied to the study of all cannabis products. Recently, a handful of companies have been able to produce products at a Good Manufacturing Practices (GMP) level which have been approved for studies addressing sleep, anxiety and chronic pain, the results of which are still years away. The inability to conduct high quality studies using plant-based products has led to a plethora of case studies and observational work. These many hurdles have considerably hindered clinical research of cannabis in Canada.

Industry support for study of medicinal effects of cannabis is lacking. Although initially committed to fostering patient care and supporting studies to examine the clinical effects of MC, the cannabis industry has renegaded on previous commitments following recreational legalization. Any promise of industry support for researcher-initiated projects has rapidly disappeared from coast to coast as the focus of these companies shifted to the rapid growth and manufacturing of non-medical GPP products.

There is reticence of patients to participate in clinical trials of MC. Given the easy access to cannabis, patients see little value to enrolling in a robust scientific study in which a placebo arm is a 50% or 33% possibility depending on the number of treatment groups. There is progressive attrition in numbers noted in cohort studies of MC (Lucas et al., 2021; Sotoodeh et al., 2022). In a Canadian cannabis producer lead cohort study, initiated prior to recreational legalization and extending into the time period of recreational legalization, the retention rate for 1,011 subjects fell to 41.4% at 6 months, with lower odds of retention following legalization (AOR 0.28, 95% CI 0.18–0.41) (Lucas et al., 2021). Similarly, an attrition rate of 75% was noted over a 12-month period for a cohort study of 323 patients with fibromyalgia followed in Canada (Sotoodeh et al., 2022).

## 4 Evolving patient acceptance of medical cannabis

Patient acceptance of MC as a treatment strategy is evolving. The changing legal status of cannabis has allowed a greater openness with more patients willing to discuss medical use or try cannabis as a treatment. The initial reticence for use can be attributed to the stigma of cannabis as a recreational drug with early studies reporting that patients had often had previous recreational cannabis experience (Ware et al., 2015; Ste-Marie et al., 2016). Following recreational legalization of cannabis in Canada, MC use among rheumatology patients has tripled according to results of an in-person clinic survey in 2014 compared to a follow-up survey in 2019 (Ste-Marie et al., 2016; Fitzcharles et al., 2020).

Policy differences between countries with legal concerns from criminalization likely continues to influence MC use (Reid, 2020). MC licenced patient numbers have however grown substantially with over 300,000 MC patients in Canada in 2020 and nearly 3 million in the US, with the majority receiving authorization for chronic pain (Boehnke et al., 2019a; Boehnke et al., 2022; Canada, 2022). Using the 2018 and 2019 National Survey on Drug Use and Health data for US subjects at least 50 years of age, past year cannabis use was at 9% for that age group, with almost one-fifth reporting medical use (Choi and DiNitto, 2021). In this study, cannabis was mostly obtained from private/informal sources and medical users were often self treating and without healthcare consultation. In a 2017 study of cannabis use over the lifespan in older adults in Colorado, 45% of 198 respondents had used cannabis in the past year, with medical need cited as reason for use for almost all new users (Arora et al., 2021).

### 4.1 Patient reported symptom relief

Chronic pain is the most common reason for Canadian patients to use MC (Walsh et al., 2013; Banerjee and McCormack, 2019; Fitzcharles et al., 2020; Meng et al., 2021). Patients’ dissatisfaction with drug treatments that are either often only modestly effective or associated with unacceptable side effects is an important driver of consideration of MC (Kosiba et al., 2019). Between 30% and 80% persons cite musculoskeletal painful symptoms as reason for MC use (Ware et al., 2005a; Swift et al., 2005; Aggarwal et al., 2009). Pain relief is also cited by patients in the US (Zaller et al., 2015; Boehnke et al., 2016; Piper et al., 2017a), United Kingdom (Ware et al., 2005b), and Australia (Swift et al., 2005) as a reason for MC use. The increased prevalence of pain in the elderly is contributing to MC use in this age group. In a survey of 470 persons over 60 years age in Colorado and Illinois, recruited *via* senior centres, health clinics and university research volunteer lists in 2017–18, almost half used some pain relieving treatment in past year with opioid use by 13%, cannabinoid use by 15%, and use of both by 15% (Bobitt et al., 2020).

Pain relief is generally reported to be substantial with use of MC (Meng et al., 2021). Numerous observational studies in various countries have reported patient satisfaction with MC for chronic pain management, including as a substitute for opioids and other pain medications (Boehnke et al., 2016; Piper et al., 2017b; Corroon et al., 2017; Boehnke et al., 2019b; Boehnke et al., 2021; Meng et al., 2021). Beyond only pain relief, surveys also report effect on sleep disturbance, although these effects have not been confirmed by randomised clinical trials (Harris et al., 2000; Nunberg et al., 2011; Ilgen et al., 2013; Walsh et al., 2013; Grella et al., 2014; Zaller et al., 2015; Lucas and Walsh, 2017; Abuhasira et al., 2018). An online survey of almost 1,000 patient members of MC dispensaries in New England rated symptom relief as 75%, with options between 0% “no relief” and 100% “complete relief” (Piper et al., 2017a). Although the numbers of patients trying MC has increased in recent years, the numbers that continue to use remain consistent at about 50% (Fitzcharles et al., 2020; Boehnke et al., 2022).

Adherence to a specific treatment over time can be seen as a surrogate for perceived efficacy. Validated real-world information about the continuation of use of MC and effect on use of other prescribed or over-the-counter medication is urgently needed. Many studies reporting favourable effects may be subject to bias due to convenience sampling or study setting in cannabis clinics or cannabis dispensary driven data. Compliance and success with use may be better when a patient is followed by a qualified healthcare professional, with guidance about products and dosing, such as reported by clinics in Israel (Abuhasira et al., 2018).

### 4.2 Demographic changes for patients using medical cannabis

Early studies of MC use in various countries reported use most commonly in younger males, with a high rate of previous recreational cannabis experience. This pattern is now changing in Canada and worldwide with increasing use by older females and without previous recreational experience (Ste-Marie et al., 2016; Fitzcharles et al., 2020; Meng et al., 2021). In the 5-year period between 2014 and 2019 in Canada, there was a shift towards more use by middle-aged women, 57% vs 78%, with mean age increasing from 53 to 61 years (Ste-Marie et al., 2016; Fitzcharles et al., 2020). Previous recreational experience with cannabis fell from over three-quarters to just under half over this time period.

Earlier studies with predominant male use show the following profile of users: 63% males, median age 45 years and 29% having previously used recreational cannabis in an Australian study in 2005 (Swift et al., 2005); and 73% males, median age 41 years, in a study of 200 users in Rhode Island in 2015 (Zaller et al., 2015); whereas males numbered 47%, mean age 49 years in a survey of 984 legal members of MC dispensaries in the US conducted in 2015–2016 (Piper et al., 2017a). These early studies prompted the question as to the true reason for MC use. Even when there is an identifiable medical condition, some may be misusing a medical diagnosis to justify use primarily for recreational reasons (Ware et al., 2005a; Swift et al., 2005).

This shift to use by older females has also been observed in the US (Yang et al., 2021). In a geriatric clinic survey in San Diego in mid 2019, 15% of 568 subjects (60% female) had used cannabis in the past 3 years, with 80% using cannabis for symptom relief with almost two-thirds having used cannabis for the first time when they were over the age of 61 years (Yang et al., 2021). In a sample of chronic pain patients, female patients had higher plasma levels of cannabidiol (CBD), cannabidiolic acid, Δ^9^-THC, and 11-hydroxy-Δ^9^-tetrahydrocannabinol, there were also specific biomarker differences between males and females. A potential sex difference in metabolizing cannabinoids, may support a difference in the effectiveness of cannabis products and sex which needs further investigation in the years ahead (Ajrawat et al., 2022).

Most US survey studies of MC use report users to be predominantly white and in recent years more likely to be employed and with higher education. In a US survey in 2016, 97% were identified as white, and 80% had an education beyond high school, and with one-third employed (Piper et al., 2017a). Some employment was reported by almost one-third in the Canadian survey done in 2014, whereas this number increased to 42% in a 2019 (Ste-Marie et al., 2016; Fitzcharles et al., 2020). Employment status and use of MC may be influenced by the legal status of cannabis, especially in the US, where it remains a federally illegal substance and workers may be subject to drug screens that do not distinguish between medical or recreational use. With more liberal laws in Canada, it can be expected that patients are less deterred by risk of litigation in the workforce, although there are restrictions for occupations such as operation of vehicles, pilots, or work in a setting requiring alert cognitive and psychomotor skills.

When personal themes regarding MC use were explored, a US study reported that cost of MC was a concern raised by over one-quarter of respondents, 11% were concerned with a stigma, 8% had difficulty accessing MC, 7% reported concerns about inhalation, and contradictions in federal and state laws was a concern for 6% of respondents (Piper et al., 2017a). Patient needs regarding cannabis were explored in focus groups of persons recruited from seniors’ centres, health clinics and cannabis dispensaries in Colorado (Bobitt et al., 2019). Lack of research and education about cannabis, lack of provider communication, challenges of access to MC, limited information on outcomes of use and a general reluctance to discuss cannabis use were identified as five main themes in that study.

## 5 The cannabis industry perspective

Although medical legalization was greeted enthusiastically by industry, it is has now become evident that the promise of immense wealth in a solely recreational market will not materialize for most companies. Although MC was strongly propelled by political agenda and governments have gained financially by taxation, this early support has declined especially with regard to funding by companies for research. As the margins related to cannabis products have decreased over time and competition with other global markets increases, it is hoped that the cannabis industry may appreciate the inherent value of formal study of high-quality proprietary formulations that could lead to regulatory approvals. There is hope that in the coming years industry will collaborate with clinical researchers to conduct high quality study of cannabis preparations in specific conditions with the aim of achieving regulatory approval and eventual health insurance coverage for the average Canadian. Industry partnerships continue to be the backbone of many academic endeavours.

### 5.1 Current Canadian cannabis market

The current cannabis market has influenced patient use of MC. MC is costly, rarely reimbursed and constitutes a considerable financial burden for patients leading some to turn to the recreational or street market. Canada has seen a progressive growth in the legal recreational market, an overproduction of cannabis and a contraction of the MC market, which now makes up only about 6% of total cannabis sales (Cannabis_market_data, 2022). In this climate of overproduction and intense competition with the illegal cannabis market, there has been a progressive reduction in price of both the legal and illegal recreational product in Canada (Stratcan, 2021; Mjbizdaily, 2022). Therefore, easier access and reduced cost are factors leading patients away from the medical route and leading to more self-administration. Another healthcare consideration being brought to the forefront is that legal medical or recreational cannabis is not regulated to the same standard as approved drugs or tobacco products. The inaccuracies of molecular concentrations on cannabis products (Oldfield et al., 2021) and contamination with levels of mold, ammonia, etc., in products being consumed in the marketplace have raised questions about lax standards at the regulatory level (Moir et al., 2008).

The increased availability of cannabis has allowed patients to bypass interaction with their healthcare provider and to self-administer. Healthcare oversight is critical when a product is used as a therapy. Patients have reported that their own personal belief in MC and physician input influences choice, with the odds of choosing MC increased 24–43 times if the physician had talked about cannabis (Bobitt et al., 2020).

### 5.2 Advantages of cannabis legalization

Progressive legalization of cannabis has some advantages. Survey studies report patient satisfaction, substantial relief for symptoms of pain, sleep disturbance and mental health symptoms, and importantly reduction of prescribed medications. Patients are now more willing to openly discuss use with health providers, although still reliant on their personal research (Boehnke et al., 2022; Holman et al., 2022). With reduced stigma of cannabis, persons without previous recreational experience are now considering use, and survey studies report use of MC for a wider range of medical conditions beyond mostly males with low back pain (Ware et al., 2015). In a recent on-line survey of over 1,500 participants with rheumatic complaints conducted in Canada and the United States, almost 50% reported current use of MC with a shift in demographics towards use by older women without previous recreational experience (IASP abstract).

## 6 Recommendations and next steps

There are a number of considerations that we believe should be emphasized to the international community regarding MC use. First, MC must be viewed similarly to any other drug by patients and the healthcare community and must follow the standard rules that apply to all prescription medications. Patients must know that MC is not a panacea free of adverse events and should therefore be authorized/prescribed (1 day) by a physician who is fully knowledgeable of the patient’s medical, mental and psychosocial condition, who can appropriately monitor for efficacy and side effects, drug-drug interactions, and who should not provide care that is solely focussed on MC use. Over the years the medical pain community has striven to move away from only drug treatment for chronic pain and promoted multidisciplinary care for patients with chronic pain.

Second, the existence of “specialized medical cannabis clinics” should be called into question. Administration of a single product is contrary to the expected standards of competent patient care. We have concerns that patients are being misled to believe that they are receiving expert care when the underlying objective is to continue the administration of MC under the auspices of a specialized clinic. Patients should also not seek medical advice from dispensary personnel who are not medically qualified.

Third, education to the public about MC must be the responsibility of the healthcare community. The reality is that patients will continue to source MC according to their individual preferences, with many choosing to continue to self-medicate. It is therefore critical that the healthcare community should be proactive in having a strong voice in the public discourse of cannabis, engage in dialogue with regulators and the cannabis industry to provide evidence-based recommendations for use of MC. It is possible that the initial enthusiasm for MC, especially when restrictions were lifted, may be more tempered with time, with a shift to a more balanced equilibrium whereby patients more critically assess the pros and cons of MC use.

Fourth, it is evident that research on cannabis will continue to differ from traditional drug research given a reliance on evidence obtained from observational and cohort studies rather than RCTs. There are many nuances that complicate research into the effects of a plant product because of the large number of molecules present with uncertainty about true effects of a specific molecule, or synergistic effects of multiple molecules termed the “entourage” effect.

Fifth, monitoring and pharmacovigilance is critical and must be supported by both governments and industry. Long term adverse effects of MC use will be critical to monitor and evaluate, especially for those younger patients using daily cannabis with anticipation for use over many years. Will there be a progressive need for increased dosing due to tolerance, will there be gradual emergence of cannabis use disorder, and will demotivation syndrome seen in recreational users also emerge for medical users? These questions will only be answered by diligent cohort and registry studies.

This overview of the evolving landscape of MC in Canada has highlighted many of the challenges that surround using cannabis as a treatment for health-related conditions. We have highlighted areas of concern regarding cannabis use in Canada and issues that need to be followed very closely in the years ahead. Central to keeping patients safe is the dissemination of accurate information guided by evidence, both of which are currently lacking. We understand that cannabis is now embedded in the fabric of Canadian society and our goal in creating this document is to stimulate discussion and hopefully course correct in the years ahead. Cannabis has the potential to become a useful tool in the armamentarium of treatments for patients dealing with chronic pain if high quality regulated products that are backed by science become available. We express the hope that MC could fill a void in effective patient care for those with chronic pain, and perhaps could become a cornerstone of treatment.
